# Supplementary material for: Poverty and Influenza/Pneumococcus Vaccinations in Older People: Data from The Survey of Health, Ageing and Retirement in Europe (SHARE) Study
Source: Vaccines (Basel). 2023 Aug 27;11(9):1422. doi: 10.3390/vaccines11091422 (PMC10534347; doi:10.3390/vaccines11091422)
Supplement: Supplementary file 1 [file vaccines-11-01422-s001.zip › vaccines-2544461-supplementary.pdf]

Supplementary Table S1. Descriptive statistics by influenza vaccination, *Poor* respondents

Variable	Influenza vaccination (n= 4,424)		No Influenza vaccination (n= 8,018)		*p*-value
Gender (female) (%)	62.66		66.67		<0.0001
Couple (%)	58.43		49.73		<0.0001
Mean age (SD)	74.27	(8.71)	70.71	(9.42)	<0.0001
Mean years of education (SD)	9.53	(4.14)	9.89	(3.78)	<0.0001
Mean household size (SD)	2.05	(1.06)	2.19	(1.31)	<0.0001
Employed/self-employed (%)	5.83		10.95		<0.0001
Make ends Meet					
1 - With great difficulty (%)	17.65		20.92		
2 - With some difficulty (%)	29.86		34.92		
3 - Fairly easily (%)	32.78		31.08		
4 - Easily (%)	19.71		13.08		
Regularly taking prescription drugs (%)	88.65		75.16		<0.0001
Self-perceived health					
1 - Excellent (%)	3.98		4.73		
2 - Very good (%)	11.48		12.40		
3 - Good (%)	43.49		43.54		
4 - Fair (%)	31.76		30.93		
5 - Poor (%)	9.29		8.41		
Mean household monthly income €	1,166.55	(727.45)	895.88	(736.35)	<0.0001
Mean number of illnesses and health conditions	1.72	(1.17)	1.45	(1.19)	<0.0001
Hip fracture (%)	3.66		2.96		
Diabetes/high blood sugar (%)	24.21		17.67		
High blood pressure/hyp. (%)	59.29		51.15		
Heart attack (%)	22.45		19.44		
Chronic lung disease (%)	10.99		7.27		
Cancer or malignant tumour (%)	7.03		5.30		
Other illness/health condition (%)	44.33		41.72		

Supplementary Table S2. Descriptive statistics by pneumonia vaccination, *Poor* respondents (excluding Romania and Slovakia)

Variable	Pneumonia vacc. (n= 1,527)		No Pneumonia vaccination (n= 10,333)		*p*-value
Gender (female) (%)	63.72		65.46		0.1864
Couple (%)	57.56		52.21		0.0001
Mean age (SD)	74.29	(8.09)	71.85	(9.41)	<0.0001
Mean years of education (SD)	10.24	(4.25)	9.71	(3.90)	<0.0001
Mean household size (SD)	1.93	(0.95)	2.14	(1.24)	<0.0001
Employed/self-employed (%)	6.35		9.63		<0.0001
Make ends Meet					
1 - With great difficulty (%)	18.34		18.80		
2 - With some difficulty (%)	23.44		34.04		
3 - Fairly easily (%)	31.04		32.65		
4 - Easily (%)	27.18		14.51		
Regularly taking prescription drugs (%)	89.98		79.05		<0.0001
Self-perceived health					
1 - Excellent (%)	4.06		4.62		
2 - Very good (%)	15.26		11.64		
3 - Good (%)	40.14		43.41		
4 - Fair (%)	30.39		31.87		
5 - Poor (%)	10.15		8.46		
Mean household monthly income €	1,272.72	(711.10)	987.80	(745.04)	<0.0001
Mean number of illnesses and health conditions	1.83	(1.21)	1.52	(1.18)	<0.0001
Hip fracture (%)	4.65		3.02		
Diabetes/high blood sugar (%)	24.75		19.40		
High blood pressure/hyp. (%)	59.40		53.31		
Heart attack (%)	23.71		20.24		
Chronic lung disease (%)	17.94		7.40		
Cancer or malignant tumour (%)	9.30		5.56		
Other illness/health condition (%)	43.09		42.88		
